# Factors associated with dysfunction of autogenous arteriovenous fistula in patients with secondary hyperparathyroidism after parathyroidectomy

**DOI:** 10.1080/0886022X.2024.2402515

**Published:** 2024-10-16

**Authors:** Boxi Chen, Qiying Fang, Yiming Tao, Siqi Peng, Shuting Deng, Ye Yuan, Nan Jiang, Sichun Wen, Bohou Li, Qiong Wu, Zewen Zhao, Pingjiang Ge, Sijia Li, Ting Lin, Zhonglin Feng, Feng Wen, Lei Fu, Zhuo Li, Jia Wen, Renwei Huang, Chaosheng He, Wenjian Wang, Guibao Ke, Lixia Xu, Shuangxin Liu, Jianchao Ma

**Affiliations:** a Department of Nephrology, Guangdong Provincial People’s Hospital (Guangdong Academy of Medical Sciences), Southern Medical University, Guangzhou, China; b Blood Purification Center of Henan Provincial People’s Hospital and People’s Hospital of Zhengzhou University, Henan Provincial Key Laboratory of Kidney Disease and Immunology, Henan Provincial Clinical Research Center for Kidney Disease, Zhengzhou, China; c Division of Otolaryngology, Guangdong Provincial People’s Hospital (Guangdong Academy of Medical Sciences), Southern Medical University, Guangzhou, China; d Department of Nephrology, The First Affiliated Hospital of Guangzhou Medical University, Guangzhou, China

**Keywords:** Hemodialysis, secondary hyperparathyroidism, parathyroidectomy, AVF dysfunction

## Abstract

**Background:**

Secondary hyperparathyroidism (SHPT) is a prevalent chronic complication in patients undergoing hemodialysis. Parathyroidectomy (PTX) is crucial for reducing mortality and improving the prognosis in the treatment of refractory hyperparathyroidism. However, it is often associated with a number of postoperative complications such as postoperative hypotension, hyperkalemia, and hungry bone syndrome. A previous study demonstrated that low blood pressure influences the patency of autogenous arteriovenous fistulas (AVF). Few studies have examined AVF dysfunction following PTX. This study aimed to identify and describe the risk variables associated with AVF dysfunction after PTX.

**Methods:**

Cases of AVF dysfunction after PTX between 2015 and 2021 were studied. Four controls were identified for each patient and were matched for sex and age. Biochemical parameters and blood pressure of the patients before and after PTX were recorded. Risk factors for AVF dysfunction after PTX were identified using conditional logistic regression analysis.

**Results:**

Sixteen patients and 64 controls were included in this study. Baseline demographic and laboratory data were compared. Patients in the AVF dysfunction group had lower levels of postoperative calcium than the controls. After surgery, calcium levels decreased more in patients with AVF dysfunction than in the control group. The decrease in systolic blood pressure (ΔSBP) after PTX was greater in the AVF dysfunction group than that in the control group. For each 1 mmHg increment in ΔSBP, the risk of AVF dysfunction after surgery increased by 11.6% (OR = 1.116, 95% CI, 1.005–1.239, *p* = .040). The likelihood of developing AVF dysfunction after surgery was twelvefold higher in diabetic patients than in non-diabetic patients (OR = 12.506, 95% CI, 1.113–140.492, *p* = .041). Among patients with ΔSBP > 5.8 mmHg after PTX, the AVF failure rate was significantly greater in patients with diabetes than in those without diabetes. Patients with a history of AVF failure had a nine-fold higher risk of developing AVF dysfunction (OR = 9.143, 95% CI, 1.151–72.627, *p* = .036). Serum albumin, hemoglobin, ΔiPTH, and age were not independent predictors of AVF dysfunction. The cutoff value for SBP was 5.8 mmHg, as determined by the Youden index of the receiver operating characteristic curve.

**Conclusion:**

Decreased systolic blood pressure (ΔSBP) after PTX, diabetes, and AVF failure history were risk factors for AVF dysfunction following PTX in patients with SHPT. Diabetes patients with ΔSBP > 5.8 mmHg were more prone to AVF dysfunction after PTX.

## Introduction

Secondary hyperparathyroidism (SHPT) is a common chronic complication in patients undergoing hemodialysis. It is characterized by disorders in intact parathyroid hormone (iPTH), serum calcium, and phosphate levels, and causes ectopic calcification, pruritus, malnutrition, and anemia. In addition, SHPT promotes bone abnormalities and cardiovascular disease, which are life-threatening conditions that negatively impact the quality of life [1]. Parathyroidectomy remains a valuable and effective treatment for severe SHPT, particularly in patients resistant to medical treatment [2, 3]. Successful parathyroidectomy can considerably reduce parathyroid hormone levels and relieve the clinical symptoms. However, it is often associated with a number of postoperative complications, including postoperative hypotension, hyperkalemia, hungry bone syndrome, hemorrhage, infection, recurrent laryngeal nerve injury, and arrhythmia [4].

The preferred vascular access for maintenance hemodialysis (MHD) patients is autogenous arteriovenous fistulas (AVF) [5], whereas central dialysis catheters and AV grafts (AVG) are less popular alternatives [6]. AVF failure in patients undergoing MHD is linked to higher rates of morbidity, mortality, and expenditure [7]. Therefore, it is essential to discover other variables that can be manipulated to prevent AVF. Diabetes mellitus, metabolic syndrome, female sex, age, vascular diameter, and hemodynamic factors have been found to be risk factors for AVF dysfunction [8–10]. Additionally, predialysis hypotension was briefly addressed in two investigations as a factor contributing to the development of AVF thrombosis. Predialysis diastolic pressure was a predictor of the risk of thrombosis in AVF in the first year after creation [11, 12]. Thrombosis can result in an AVF failure. However, aside from mechanical interventions, there are currently very few available medical treatments that might improve the long-term viability of AVF [13]. Most studies have focused on AVF maturation or AVF dysfunction in the long-term follow-up period. However, little is known regarding AVF dysfunction following PTX treatment. Therefore, this study aimed to identify the risk factors associated with AVF dysfunction after PTX.

## Materials and methods

### Study population

The eligibility criteria were (I) age ≥18 years and ≤ 75 years. (II) Patients who received MHD treatment for ≥3 months, three times a week for 4 h each time. (III) Severe SHPT is associated with severe bone pain, itchy skin, osteoporosis, myalgia, and other symptoms that negatively impact quality of life. (IV) Hypercalcemia or hyperphosphatemia resistant to medical treatment. Persistent iPTH ≥ 800 pg/mL resistant to medical treatment. (V) Parathyroid gland hyperplasia is seen on imaging (>1.0 cm in diameter). (VI) Patients with AVF dysfunction after PTX. Exclusion criteria: (I) History of malignancy. (II) Patients cannot expose the surgical area of the neck because of severe bone deformities. (III) Patients who cannot endure general anesthetic surgery because of serious heart, lung, or brain malfunctions. (IV) Patients with severe infection, hypoproteinemia (blood albumin < 30 g/L), or coagulation dysfunction. (V) Uncontrolled severe hypertension.

### Surgical procedure

Before receiving PTX, all patients underwent hemoddialysis within a 24-h period. Subsequently, a skilled surgeon performed the surgical procedures in the Otolaryngology Head and Neck Surgery Department at Guangdong Provincial People’s Hospital. The surgical procedure was as follows. After general anesthesia, the low-collar incision length was 6–7 cm. Once the recurrent laryngeal nerve was located, the inferior and superior parathyroid glands were removed. The last hyperplastic parathyroid gland was selected and dissected into pieces for implantation into the surface of the brachioradialis muscle of the arm. To confirm the diagnosis, all resected specimens were sent to the pathology laboratory. During the operation, the patient’s arm side of the arteriovenous fistula is placed in the appropriate position to avoid accidental extrusion caused by the doctor. Calcium supplements were administered intravenously to all the patients after PTX to prevent postoperative hypocalcemia. The formulae created in our previous study were used to calculate the dosage of calcium supplement [14].

### Data collection

All data were retrospective. Medical data included the duration of dialysis, duration of SHPT, history of diabetes and hypertension, and antihypertensive medications. Each patient’s blood pressure was monitored and recorded 30 min before and after surgery by a single vascular access research nurse. The patient lay flat when the nurse measured the blood pressure. The average of the three preoperative readings and the average of the three postoperative readings were recorded using an automated monitor.

The following data were collected: hemoglobin, serum albumin, platelets, hematocrit (HCT), serum blood creatinine (Cr), serum blood urea nitrogen (BUN), serum calcium, serum phosphorus, serum alkaline phosphatase (ALP), and intact parathyroid hormone (iPTH).

AVF dysfunction is characterized as localized stenosis of more than 50% of the normal vessel diameter in the vicinity accompanied by the following: natural blood flow of the endovascular fistula <500 mL/min; inability to meet the blood flow requirements of the dialysis prescription [15]. And the access dysfunction was considered within 2 weeks after surgery. We evaluated arteriovenous fistula by the blood flow of the arteriovenous fistula.

Patients with AVF dysfunction were paired at a ratio of 1:4 and compared to controls. Patients who underwent AVF dysfunction after PTX were identified between 2015 and 2021 at Guangdong Provincial People’s Hospital. The study protocol was approved by the Institutional Ethics Board of Guangdong Provincial People’s Hospital (GDREC2019512A), and all participants provided informed consent.

### Statistic analysis

The mean ± SD (range) or interquartile range was used to present descriptive data. For data with a normal distribution, an independent sample t-test was used to compare continuous variables, and for data with a non-normal distribution, the Mann-Whitney test was used. Categorical variables were described using frequency counts and percentages, and the chi-square test or Fisher’s exact test was used to assess the statistical differences between the two groups. The connections between ΔSBP and various factors were investigated using Pearson or Spearman correlation coefficients. Independent risk variables for postoperative AVF dysfunction were found using binary conditional logistic regression analysis. The covariates selected for inclusion in the multivariate models were determined by (1) the potential to confound the association between the occurrence of AVF dysfunction and other risk factors, (2) the biological plausibility of an association with the occurrence of AVF dysfunction, and/or (3) the magnitude of their univariate relationship with the presence of AVF dysfunction. Predictive parameter values for AVF dysfunction were calculated from the receiver operating characteristic (ROC) curves. Statistical significance was assigned to variables with a *p*-value < .05. SPSS version 26.0 for Windows was used for all data processing.

## Results

**Figure 1.**
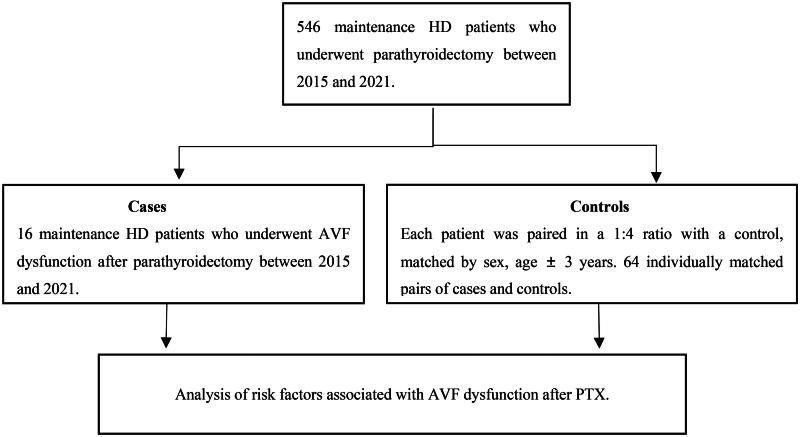
Patients recruitment process.

In our study, 546 maintenance hemodialysis patients underwent parathyroidectomy between 2015 and 2021. AVF was used in 410 patients, catheters in 82 patients, and AVG as vascular access in 54 patients. The number of participants in each phase of the study is shown in Figure 1. For the final analysis, 16 individually matched pairs of cases and 64 controls were included. The fistula was located on the right side in 15 patients, and on the left side in 65 patients. The baseline demographic and laboratory data of the patients and controls are presented in Table 1. The duration of dialysis, history of hypertension, preoperative serum calcium level, and preoperative iPTH level were similar between the two groups. The mean age of the AVF dysfunction group was 49.0 (38.5–53.0) and was compared to controls 49.0 (39.0–53.0), as expected given age-matching. The patients were more likely to have diabetes mellitus and were taking β-blocker medications. Compared with controls, patients with AVF dysfunction exhibited lower postoperative serum calcium levels. After surgery, serum calcium levels decreased to a greater extent in patients with AVF dysfunction than in the control group. There were no appreciable differences in preoperative systolic blood pressure between the two groups. The decrease in systolic blood pressure after PTX was greater in the patients with AVF dysfunction than in the control group.

**Table 1.** Demographics and clinical characteristics of the study population.

Characteristics	Cases	Controls	*p*-value
	*n* = 16	*n* = 64	
Age (years)	49.0(38.5,53.0)	49.0(39.0,53.0)	.928
Weight (kg)	58.1 ± 11.5	54.4 ± 9.9	.200
AVF failure history	6(37.5%)	8(12.5%)	.019
Duration of dialysis (years)	6.3 ± 3.4	7.5 ± 3.0	.180
Duration of SHPT (months)	9.5(6.0,21.3)	12.0(6.0,24.0)	.815
Comorbidity (*n*, %)			
Diabetes mellitus	6(37.5%)	2(3.1%)	.000
Hypertension	9(56.3%)	42(65.6%)	.485
Hemoglobin (g/L)	107.0 ± 23.4	104.7 ± 18.5	.668
Albumin (g/L)	38.6 ± 4.2	37.7 ± 4.1	.400
Platelet (×10^9^/L)	225.0(175.8,262.3)	204.0(169.0,246.5)	.470
HCT	0.3 ± 0.1	0.3 ± 0.1	.858
Preoperative Cr (μmol/L)	875.3(757.0,1219.2)	863.8(738.1,1068.4)	.622
Preoperative BUN (mmol/L)	22.3 ± 7.0	23.4 ± 7.5	.589
Antihypertensive drugs (*n*, %)			
ACEI/ARB	5(31.3%)	22(34.4%)	.813
CCB	8(50.0%)	38(59.4%)	.497
β-blocker	11(68.8%)	26(40.6%)	.044
α-blocker	4(25.0%)	14(21.9%)	.789
Preoperative SBP (mmHg)	149.3 ± 22.7	144.0 ± 21.0	.372
Postoperative SBP (mmHg)	135.7 ± 24.1	143.4 ± 21.1	.213
Preoperative DBP (mmHg)	85.5(81.2,91.5)	87.3(78.3,93.6)	.741
Postoperative DBP (mmHg)	80.2 ± 11.7	79.8 ± 10.2	.876
ΔSBP (mmHg)	11.0(5.3,24.1)	1.8(−3.9,6.2)	.001
ΔDBP (mmHg)	6.5 ± 9.5	6.0 ± 8.1	.840
Preoperative ca (mmol/L)	2.5 ± 0.2	2.5 ± 0.2	.770
Postoperative ca (mmol/L)	2.1 ± 0.3	2.3 ± 0.3	.007
ΔCa (mmol/L)	0.5(0.3,0.6)	0.2(0.0,0.3)	.003
Preoperative phosphorus (mmol/L)	2.2 ± 0.5	2.4 ± 0.6	.170
Postoperative phosphorus (mmol/L)	1.7 ± 0.5	1.7 ± 0.4	.937
Preoperative ALP (U/L)	498.5(224.0,716.3)	294.0(167.8,574.8)	.118
Postoperative ALP (U/L)	503.0(148.3,650.3)	277.0(152.8,507.5)	.238
ΔALP (U/L)	26.0(−14.3,85.3)	11.5(−8.8,54.0)	.568
Preoperative iPTH (pg/mL)	1825.5(1208.0,2234.8)	1888.0(1320.3,2495.8)	.413
Postoperative iPTH (pg/mL)	29.5(17.9,47.2)	45.2(22.6,93.1)	.184
ΔiPTH (pg/mL)	1766.3(1071.9,2111.2)	1826.2(1175.4,2395.5)	.354

SBP: systolic blood pressure; DBP: diastolic blood pressure; Cr: creatinine; HCT: hematocrit; BUN: blood urea nitrogen; iPTH: intact parathyroid hormone; ALP: alkaline phospharase; Δ: difference between preoperative and postoperative; Groups were significantly different, *p* <.05.

**Figure 2.**
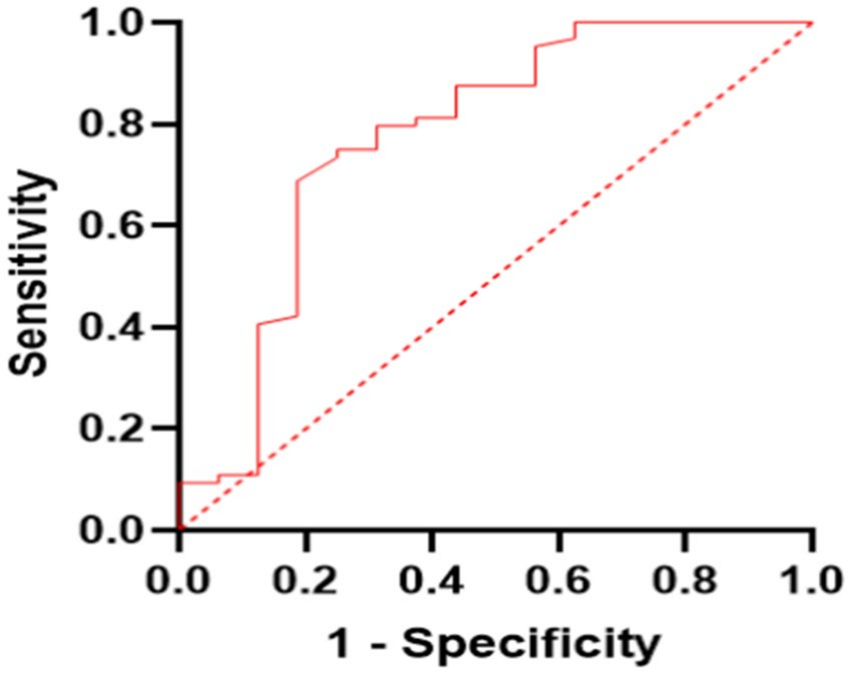
ROC curve for postoperative AVF dysfunction by the conditional logistic regression. AUC for ΔSBP was 0.775, *p* = .001. The cutoff value for ΔSBP was 5.8 mmHg. The sensitivity and specificity of ΔSBP were 75.0% and 75.0%.

Table 2 displays the relationship between the risk variables and likelihood of AVF dysfunction. An increased likelihood of developing AVF dysfunction was linked to a higher drop in systolic blood pressure after surgery (odds ratio [OR] = 1.116, 95% confidence interval [CI], 1.005–1.239, *p* = .040). In light of this, the cutoff point for systolic pressure difference was chosen in the early prediction of AVF dysfunction following surgery according to the area under the curve (AUC) and the highest Youden index based on the ROC curve. The cutoff point (5.8 mmHg) of the systolic pressure difference may predict AVF dysfunction after surgery (*p* < .05) (Figure 2). When we used 5.8 mmHg as the cutoff point for systolic blood pressure difference, the incidence of AVF dysfunction after surgery was higher with a greater SBP difference than a lesser SBP difference (AUC = 0.775, *p* = .001). The likelihood of developing AVF dysfunction after surgery was twelvefold higher in diabetic patients than in non-diabetic patients (OR = 12.506, 95% CI, 1.113–140.492, *p* = .041). Diabetes is a risk factor associated with AVF dysfunction following PTX in patients with SHPT. We further investigated the relationship between AVF failure rate and ΔSBP in individuals with diabetes. The AVF failure rate after PTX in patients with ΔSBP ≤ 5.8 mmHg was not related to the presence of diabetes, whereas patients with ΔSBP > 5.8 mmHg with diabetes had a significantly greater AVF failure rate after PTX than patients without diabetes (Table 3). Patients with a history of AVF failure were at a ninefold higher risk of developing AVF dysfunction (OR = 9.143, 95% CI, 1.151–72.627, *p* = .036). Older age and higher platelet counts were not associated with an increased likelihood of AVF dysfunction.

**Table 2.** Conditional logistic regression analysis of the association between risk factors and postoperative AVF dysfunction.

Covariate	Univariate regression		Multivariate regression	
	OR (95%CI)	*p*-value	OR (95%CI)	*p*-value
ΔSBP (mmHg)	1.107 (1.032–1.188)	.004	1.116 (1.005–1.239)	.040
Preoperative iPTH (pg/mL)	1.000 (0.999–1.000)	.262	0.999 (0.998–1.001)	.372
Duration of dialysis (years)	0.874 (0.719–1.062)	.175		
ΔiPTH (pg/mL)	1.000 (0.999–1.000)	.238		
Age (years)	1.782 (0.589–5.389)	.306		
Weight (kg)	1.040 (0.983–1.101)	.173		
Platelet (×10^9^/L)	1.001 (0.992–1.009)	.886		
Preoperative Cr (μmol/L)	1.001 (0.998–1.003)	.545		
Preoperative BUN (mmol/L)	0.977 (0.902–1.058)	.568		
Preoperative SBP (mmHg)	1.012 (0.985–1.039)	.384		
Aortic calcification	5.376 (1.087–26.585)	.039		
AVF failure history	5.087 (1.210–21.391)	.026	9.143 (1.151–72.627)	.036
Preoperative ca (mmol/L)	1.522 (0.101–22.985)	.762		
Albumin (g/L)	1.072 (0.924–1.243)	.357		
Hemoglobin (g/L)	1.007 (0.978–1.037)	.658		
Hypertension	0.672 (0.219–2.063)	.487		
Diabetes mellitus	21.662 (2.585–181.526)	.005	12.506 (1.113–140.492)	.041

SBP: systolic blood pressure; iPTH: intact parathyroid hormone; BUN: blood urea nitrogen; Cr: creatinine; Δ: difference between preoperative and postoperative; OR: odds ratio; *p* < .05.

**Table 3.** Comparison of AVF failure rate after the PTX in SHPT patients with or without diabetes.

Diabetes	ΔSBP > 5.8 mmHg		ΔSBP ≤ 5.8 mmHg	
	cases, *n*(%)	AVF failure number, *n*	cases, *n*(%)	AVF failure number, *n*
Yes	7(25.0)	6	1(1.9)	0
No	21(75.0)	6	51(98.1)	4
*p* value	.027		1.000	

SBP: systolic blood pressure; *p*<.05.

Sixteen patients with AVF dysfunction after PTX were identified. Eleven patients underwent percutaneous transluminal angioplasty after fistula failure. Two patients underwent thrombolysis therapy and three patients underwent AVF reconstruction. Three patients experienced AVF dysfunction after 12 months. The AVF blood flow of the remaining patients met the hemodialysis treatment criteria.

In a correlation study with ΔiPTH, age, dialysis duration, and preoperative systolic blood pressure as independent variables, no associations were detected between ΔSBP and any of them. However, the calcium level difference seemed to be an association variable of ΔSBP (*r* = 0.381, *p* = .001) (Table 4).

**Table 4.** Univariate analysis of ΔSBP and parameters by correlation analysis.

Parameters	ΔSBP	
	R	*p*-value
Dialysis duration (years)	−0.093	.412
ΔCa (mmol/L)	0.381	.001
ΔiPTH (pg/mL)	0.096	.398
Preoperative SBP (mmHg)	0.169	.134
Age (years)	0.134	.235
Preoperative DBP (mmHg)	0.042	.711
Hemoglobin (g/L)	−0.067	.552
ΔDBP (mmHg)	0.329	.003
Platelet (×10^9^/L)	0.132	.243
Weight (kg)	0.063	.580
ΔALP (U/L)	0.087	.441

SBP: systolic blood pressure; iPTH: intact parathyroid hormone; ALP: alkaline phospharase; DBP: diastolic blood pressure; Δ: difference between preoperative and postoperative; *p*<.05.

## Discussion

In the last decade, as chronic kidney disease (CKD) has become increasingly common, the demand for hemodialysis has increased [16]. AVF is the optimal choice for hemodialysis. However, the long-term patency rate was only marginally acceptable [17]. Few studies have focused on AVF dysfunction after PTX. In view of the importance of AVF in the maintenance hemodialysis patients, it is worthwhile to further investigate the risk factors for AVF dysfunction after PTX. The current results showed that a decrease in systolic blood pressure after surgery, diabetes, and history of AVF failure were independent risk factors for AVF dysfunction after PTX.

It is important for hemodialysis patients to manage their blood pressure. Intradialytic hypotension, which is common in patients undergoing maintenance hemodialysis, may be associated with higher mortality. Hypotension after parathyroidectomy is associated with occlusion of the arteriovenous fistula. In this case-control study, we discovered that a decrease in systolic blood pressure acts as a risk factor for AVF dysfunction following PTX. An increased likelihood of developing AVF dysfunction is associated with a greater decrease in systolic blood pressure after surgery. The development of neointimal hyperplasia or thrombosis around fistula anastomosis is one of the most common reasons for lumen narrowing and AVF failure is the development of neointimal hyperplasia or thrombosis around the fistula anastomosis [18]. Hemodialysis access thrombosis is a complex process that causes fistula occlusion and the loss of dialysis function. Furie B et al. proposed three elements of thrombosis: blood flow, coagulation components, and blood vessel wall [19]. The hypercoagulable state and increase in thrombotic antibodies, platelet factors, and endotheliocyte receptors can be caused by uremia. In the presence of an AVF, thrombosis develops because of wall shear stress and altered AVF flow [20]. In a prospective study involving 170 dialysis patients, May RE et al. found that the risk of thrombosis increased with decreasing access blood flow, as monitored by ultrasound dilution techniques [21]. These factors can initiate the thrombotic cascade and combine to increase its potency. A high percentage reduction in SBP was associated with low blood flow in the AVF, and AVF dysfunction may be caused by fistula thrombosis due to low blood flow. Therefore, management of blood pressure is crucial. Using ROC curve analysis, we found a statistically significant cutoff point for systolic blood pressure difference that predicts postoperative AVF dysfunction. A systolic blood pressure difference > 5.8 mmHg is associated with a higher risk of AVF failure in hemodialysis patients after PTX. Consistent with previous reports, there was a positive association between the decrease in calcium (Δca) and the change in systolic blood pressure (ΔSBP) following surgery [22]. Nilsson et al. discovered that acute hypercalcemia results in increased systolic blood pressure and impaired endothelial vasodilatory function [23]. Increased serum calcium may cause vasoconstriction by increasing calcium levels in smooth muscle cells of the vascular system, thus regulating blood pressure. In addition, a case series demonstrated that three patients with SHPT experienced long-lasting and severe hypotension immediately after parathyroidectomy. Meanwhile, postoperative calcium levels dropped abruptly [24]. These data suggest that changes in serum calcium levels may cause changes in blood pressure.

In our study, univariate analysis find that aortic calcification was an influencing factor for arteriovenous fistula failure, and the percentage of aortic calcification was higher in the arteriovenous fistula failure group. At the same time, blood pressure and blood calcium have a certain correlation, and elevated blood calcium can increase blood pressure. However, the differences in preoperative blood pressure and preoperative calcium between the arteriovenous fistula failure group and the normal arteriovenous fistula group were not statistically significant. Aortic calcification is a long-term process, elevated blood pressure constantly impacts the vessel wall, damaging the vessel intima, which then triggers scar-like hyperplasia of the vessel wall. At the same time, elevated blood calcium causes calcium salts to be deposited on the vessel wall constantly, aggravating the aortic calcification, and ultimately leading to lumen narrowing.

Our study demonstrated a higher risk of AVF dysfunction after PTX in diabetic patients. Previous studies indicated that diabetes is associated with AVF dysfunction. Hayakawa et al. observed that diabetes is a risk factor for successfully maintaining vascular access, which is consistent with our findings [25]. Gheith et al. validated these findings, indicating that vascular access survived longer in non-diabetic individuals than in diabetic ones [26]. Abnormal glucose homeostasis is associated with increased oxidative stress, increased cell proliferation, and impaired endothelial function [27–29]. Diabetes has been shown to have a significant impact on AVF dysfunction by worsening systemic inflammation and macrovascular involvement. Secondary hyperparathyroidism is less common in diabetic dialysis patients. At the same time, diabetic patients with dialysis have a higher incidence of cardiovascular disease than other dialysis patients. When progressing to the stage of severe secondary hyperparathyroidism, patients have reduced cardiac function and cannot tolerate surgery. Also, diabetic patients have a poorer prognosis, which could explain the smaller proportion of diabetes dialysis in our study.

In our study, we further found that diabetic patients with ΔSBP > 5.8 mmHg were more prone to AVF dysfunction after PTX. A possible cause of arteriovenous fistula occlusion is the combination of thrombus and damaged vascular intima. The AVF failure rate after PTX in patients with ΔSBP ≤ 5.8 mmHg was not related to the presence of diabetes. This may indicate that the main risk factor is a decrease in blood pressure during AVF failure.

The main limitation of our study was its small sample size, which limited our ability to investigate additional risk factors. We were unable to analyze additional risk factors for AVF dysfunction after PTX. Further research with larger sample sizes is required to identify new risk factors. In addition, the study was a retrospective analysis; therefore, selection bias was inevitable. Moreover, this was a single-center study; thus, the findings may not be relevant to dialysis patients at other facilities. Finally, many of the individuals in our study had diabetes, and we only recorded their supine blood pressure. We did not consider the possibility that they experienced orthostatic hypotension as a result of autonomic dysfunction, which could have been a confounding factor for AVF failure in this study.

In conclusion, hemodialysis patients with AVF dysfunction following parathyroidectomy may have higher mortality and rehospitalization rates. Findings from this study implicated a decrease in systolic blood pressure after surgery, diabetes, and AVF failure history as independent risk factors for AVF dysfunction. A reduction in SBP was associated with a decrease in serum calcium. Based on these observations, it is essential to monitor the blood pressure and serum calcium levels during the perioperative period. For high-risk patients with significant postoperative blood pressure reduction, we suggest that experienced clinicians use ultrasound technology to monitor arteriovenous fistula blood flow. A prospective controlled multicenter study will help us better explore the risk factors for AVF failure after PTX and prevent this condition.

## Supplementary Material

Ethics approval form.pdf
