# Supplementary material for: Factors associated with dysfunction of autogenous arteriovenous fistula in patients with secondary hyperparathyroidism after parathyroidectomy
Source: Ren Fail. 2024 Oct 16;46(2):2402515. doi: 10.1080/0886022X.2024.2402515 (PMC11486174; doi:10.1080/0886022X.2024.2402515)
Supplement: Ethics approval form.pdf [file IRNF_A_2402515_SM0364.pdf]

Guangdong Provincial People' s Hospital (Guangdong Academy of Medical Sciences)

Medical Research Ethics Committee

No.GDREC2019512H(R1)

Approval Letter of the Research Ethics Committee

Study title	Clinical and basic study of parathyroidectomy in hemodialysis patients with secondary hyperparathyroidism			
Principal investigator	Shuangxin Liu	Project No. And Issued BY	"Summit Plan" clinical and basic integration project	
Reviewed items	Protocol No.	20190613	Protocol dated	2019.06.13
	Informed consent form NO.	1.0	Informed consent form dated	2019.07.09
	Other specify	Authorization duty sheet.		
Types of review	Review quickly after revision	Number of reviewers	13	
Evaluation results	● Approved<br>○ Approved after revision<br>○ Re-reviewed after revision<br>○ No approval<br>○ Suspension or Termination of the approved research<br>* Comments in the attachment			
Signature Chair:		Date of review:	2019年08月10日	

广东省人民医院 医学研究伦理委员会

Note:1.In order to fully guarantee the rights and interests of the study subjects, if there is any change in the study protocol, informed consent and recruitment materials, or if there is any serious adverse event (human body) or other unexpected situation after the start of this trial, please report to the Committee immediately. The Committee has the right to terminate the trial.

2.Projects with a study duration of more than one year should submit an annual progress report to the Ethics Committee for follow-up review.

3.At the end of the study, please submit the final report to the Ethics Committee for review.

The Committee operates in accordance with ethical principles such as the《 Declaration of Helsinki 》and the requirements of national regulations.
